# No laughing matter: Latinas’ high quality of conversations relate to behavioral laughter

**DOI:** 10.1371/journal.pone.0214117

**Published:** 2019-04-10

**Authors:** Nairán Ramírez-Esparza, Adrián García-Sierra, Gloriana Rodríguez-Arauz, Elif G. Ikizer, Maria J. Fernández-Gómez

**Affiliations:** 1 Department of Psychological Sciences, University of Connecticut, Storrs, Connecticut, United States of America; 2 Department of Speech, Language and Hearing Sciences, University of Connecticut, Storrs, Connecticut, United States of America; 3 School of Psychology, University of Costa Rica, San José, San Pedro, Costa Rica; 4 Department of Psychology, Yale University, New Haven, Connecticut, United States of America; 5 Department of Psychology, Indiana University of Pennsylvania, Indiana, Pennsylvania, United States of America; University of Colorado Denver, UNITED STATES

## Abstract

Latinx in the United States have greater life expectancy than other groups, in spite of their socioeconomic and psychosocial disadvantage. This phenomenon has been described as the Latinx health paradox. This investigation observed the interplay of cultural processes and social networks to shed light on this paradox. Latina (N = 26) and White-European (N = 24) mothers wore a digital recorder as they went about their daily lives. Four conversation styles were characterized from the recordings to measure the mothers’ quality of their conversations (small talk and substantive conversations) within different social networks (with the father vs. other adults). As a positive indicator of well-being, laughter was assessed during the conversations. Results demonstrated that Latina mothers tend to laugh more than White-European mothers; and that this relation is mediated by substantive conversations with others. This suggests that Latinas’ cultural processes afford meaningful conversations, which relates to more behavioral laughter, a process that may have positive implications on well-being.

## Introduction

Latinxs living in the United States have a greater life expectancy than other groups (including non-Hispanic Whites), despite their socioeconomic and psychosocial disadvantages. This phenomenon is commonly known as the Latinx health paradox [1]. Cultural processes may explain health advantages among Latinos /as. For example, in general Latinxs are collectivistic and prefer to be with others rather than alone; they are talkative and gregarious; and they enjoy interacting with family and friends [2, 3]. In addition, Latinxs cherish the cultural values such as *Simpatía–*because they value positive social interactions by being respectful and polite [3–6]; and *familism* (also termed familialism or familismo)—because they value family loyalty and view the family as a source of instrumental support [7, 8]. Accordingly, these cultural processes promote greater social integration and tighter social connections with others, which relate to health outcomes (e.g., lower risk for mortality, lower stress and anxiety [9, 10]).

**Fig 1:**
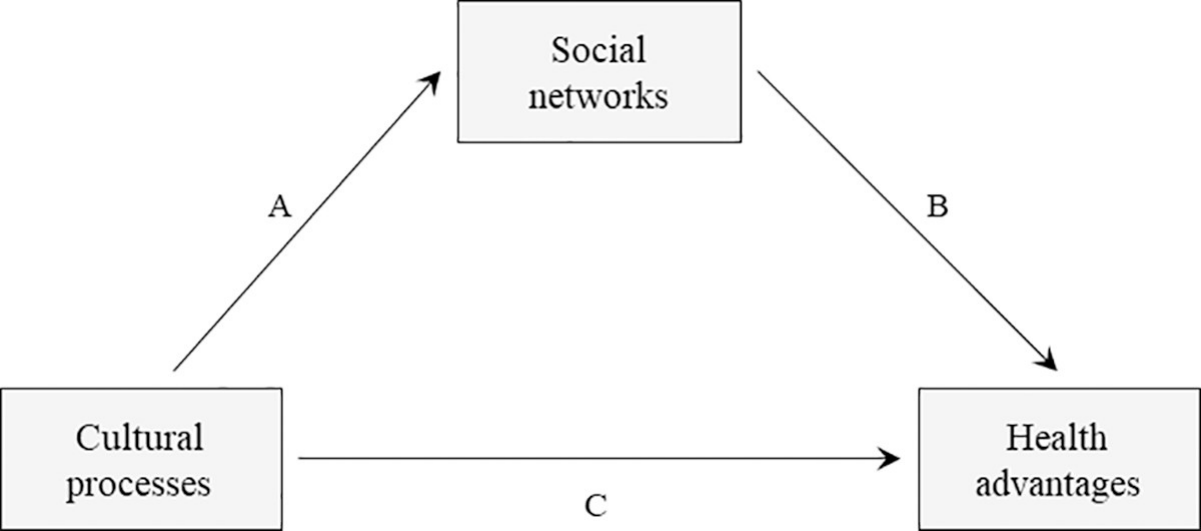
The sociocultural health resilience model. This adapted figure from Ruiz and colleagues [1] illustrates how social networks (family, community) mediate the association between Latinxs’ cultural processes and health advantages.

Ruiz and colleagues [1] provide a sociocultural resilience model to explain the interplay of Latinxs’ cultural processes (e.g., collectivism, familism), social networks (i.e., social resources, such as friends, family, and other acquaintances), and health outcomes. Their model suggests that cultural processes relate to health advantages, and that social networks mediate this relationship (see Fig 1 adapted from Ruiz and colleagues [1]).

### Sociocultural resilience model

#### Cultural processes and social networks (Path A, Fig 1)

Ruiz and colleagues [1] propose that Latinxs’ cultural processes are associated with different social network characteristics that distinguish them from the White-European American culture. Latinxs are collectivistic and value social interactions with others [2, 3]. Latinxs also value familism, which suggests that Latinxs have strong identification and attachment with their nuclear family and extended families [7–9]. For example, Valenzuela and Dornbusch [11] asked Mexican-origin and White-European adolescents living in the United Stated to indicate the number of adult relatives living within an hour’s drive of the respondent’s home on a six-point scale from “none” to “20 or more.” The results showed that Mexicans-origin adolescents reported between six and ten adult relatives living close to home, as opposed to between three and five among White-European adolescents. Similarly, Almeida and colleagues [12] compared foreign-born Latinx, U.S.-born Latinx and non-Latinx white samples in terms of perceived social support from friends and family. The authors found that foreign-born Latinxs were more likely to perceive higher social support compared to the other samples and this difference remained significant even after controlling for socioeconomic status.

Latinxs show this association between their cultural processes and their socialization with others in their everyday natural behaviors as well. For example, Ramírez-Esparza and colleagues [2] asked undergraduate students living in Mexico and undergraduate student living in the United States to wear a digital recorder to capture snippets of ambient sounds as they went about their lives. The authors then asked coders to identify different social interaction categories and the results demonstrated that cultural processes were associated with different forms of socialization across the groups. Specifically, Mexicans, in accordance with collective values, spent more time in face-to-face interactions (both in dyads and in groups), whereas White-European Americans, in accordance with individualistic values, spent more time having remote interactions (e.g., on the phone) and more private interactions (e.g., in their home as compared to public places).

The above mentioned studies inform about the association between cultural processes and social networks by using ethnicity (i.e., the cultural group individuals identified with) as a proxy of cultural processes. However, other studies provide evidence for this association by including actual assessments of cultural processes. For example, Campos and colleagues [9] found that self-reported familism among Latinxs living in the U.S. is strongly associated with higher perceived social support, than among individuals from European backgrounds. In a more recent study, Campos, Rojas Perez and Guardino [13] compared a sample of Latinxs with samples from European and East-Asian backgrounds and found that only among the Latinx sample, self-reported familism was associated with higher romantic relationship quality through an association with more secure attachment.

#### Social networks and health (Path B, Fig 1)

Ruiz and colleagues [1] provide evidence for the association between social networks and health outcomes, including other psychosocial indicators of health and well-being. It is well-known that social support buffers the effects of psychological stress on a diverse range of health outcomes [14]. For example, research has shown that lower levels of support following a heart attack are associated with a greater risk of subsequent cardiac morbidity and mortality [15]. Furthermore, social support is associated with lower levels of stress and lower physical pain [16], and lower systolic blood pressure [17]. Close relationships (e.g., with friends and acquaintances) have also been shown to have a positive effect on health [18]. Ruiz and colleagues [1] argue that although most of the studies that have tested the relation between social networks and health outcomes do not speak directly to the relation between culture and health, they do leave open the possibility that social networks can be a common mediational pathway.

#### Cultural processes and health (Path C, Fig 1)

Finally, Ruiz and colleagues [1] propose that there is an association between cultural processes and health. That is, values like familism and collectivism lead to better health outcomes. Studies that have used ethnicity as a proxy for cultural processes have shown that Latinxs as a group experience better health and live longer than non-Latinxs [1, 19]. Latinxs’ cultural processes have been also associated with other behavioral and psychological indicators of health, such as well-being. Bowman [20] conducted a study with a diverse sample of freshman college students from colleges and universities across the U.S. and found that being Latinx was associated with greater well-being upon entering college. Adams and Boscarino [21] found that Hispanics were less likely than their White counterparts to develop symptoms of major depression in the aftermath of the September 11^th^ attacks, after controlling for meaningful variables such as individual stressors. At the macro level Latin American countries exhibit higher levels of perceived well-being, compared to Non-Latin countries of similar income level [22].

Other relevant studies have assessed well-being in combination with actual cultural processes. For example, Campos and colleagues [9] found that pregnant Latina women in comparison with White-European pregnant mothers reported higher familism; and familism contributed to better social support, as well as reduced stress and pregnancy anxiety. Similarly, Schwartz and colleagues [23] carried out a study that included participants who identified themselves as White-European American and participants who identified themselves from other ethnic groups (e.g., Latinxs, Blacks, Asians). The results showed that although White-Europeans endorsed collectivistic values to a lesser extent than other ethnic groups, they also benefited from endorsing collectivism. That is, across ethnic groups, collectivist values was associated with well-being outcomes.

#### Summary

There is evidence that Latinxs’ cultural processes lead to higher social cohesion and stronger social networks, which both arguably derive from distinct cultural processes relative to White-European Americans. There is further evidence that both being a Latinx and having more cohesive social networks are each associated with more positive physical health and well-being outcomes. The sociocultural resilience model suggests that stronger social networks have a positive mediating influence on these health outcomes, ultimately stemming from the influence of cultural processes. A couple of published studies lend support to the interplay of variables that fit within the sociocultural resilience model.

Campos and colleagues [24] assessed as cultural process, familism in White-European, Latinx, and Asian samples, and as social networks, the authors used assessments on perceived closeness with family members, and social support. Finally, as a health outcome they included an overall assessment of psychological health (i.e., a combination of perceived stress, general mental health, and depressive symptoms). The results showed that as expected Latinxs scored higher in familism; however, all groups benefited from endorsing more familism. Specifically, higher familism was indirectly associated with higher psychological health via a pathway through both; higher perceived closeness with family members, and higher perceived social support. In a recently published study, Campos and colleagues [25] found that familism buffered cortisol responses via higher perceived social support. In this study, however, this association was only beneficial for Latinxs as compared to other ethnic groups (i.e., White-European, and Asians).

The Latinx health paradox is widely supported in the literature; however, as cultural scientists point out, the understanding of the interpersonal/cultural mechanisms that contribute to this phenomenon are still in its beginnings [1, 26, 27]. In this study, we sought to provide additional evidence in support of the sociocultural resilience model. Specifically, we compared Latina mothers with White-European mothers in terms of the quality of the conversations they have in their natural environments. We also examined how the quality of these interactions relate to one specific psychosocial indicator of well-being: behavioral laughter, as it occurs in natural environments. Although we did not examine a physical health outcome, we propose here that laughter is a behavioral indicator of, or precursor to, well-being analogous to other psychological indicators highlighted by Ruiz and colleagues’ sociocultural resilience model (e.g., stress, smoking). In other words, smoking can be regarded as a behavior that leads to poor health, but it is not a physical assessment of physical or psychological health in and of itself. Likewise, laughter is a behavior that can lead to a positive sense of well-being.

### Laughter as a psychosocial positive indicator of well-being

Laughter has been associated with psychophysiological health, well-being, and relational advantages in different social contexts. Regarding the association between laughter and psychophysiological health, Berk [28, 29] provides an overview of the research and indicates that laughter has been linked to an increase in pain threshold, breathing, relaxation of muscle tension, and improved indicators of mental functioning such as alertness, creativity, and memory.

Regarding the effects of laughter on psychological well-being, Bonnano and Kelter [30] interviewed 38 recently widowed women and found that expressions of positive emotion such as laughter predicted decreased grief after 25 months of conducting the interview. In a follow-up study Bonnano and colleagues [31] found that among participants that presented a history of childhood sexual abuse, those that expressed positive emotion indicators such as laughter when describing a non-abuse past event presented improved social adjustment at a two-year follow-up. However, laughter was not always conducive to well-being; when laughter happened while describing a past episode of abuse, it was not related to increased well-being in the participants.

Research has also shown that laughter is associated with positive relational outcomes in different social contexts. For example, Kurtz and Algoe [32] explored the role that shared laughter has on the well-being of romantic couples’ relationships. They used a detailed coding scheme for the number of times that 71 heterosexual couples laughed together when talking in a video-taped lab session about how they first met. They found that these instances of shared laughter were positively and uniquely related to increased reports of relationship well-being (e.g., relationship quality, closeness and social support) above and beyond other laughter that occurred during the interaction. In a related study, Kashdan, Yarbro, McNight, and Nezlek [33] explored the role of laughter as a “social booster” in the natural environment. They conducted a two-week daily diary study where they asked 162 participants to recall their face-to-face social interactions in each day and to record whether they laughed and whether they experienced a variety of outcomes including positive emotions after the encounter in which laughter occurred. The authors found that laughing with another person during an interaction was uniquely related to greater intimacy, positive emotions, and enjoyment in subsequent interactions.

What is particularly interesting about these last two studies is the novel methods that researchers used to measure laughter (i. e., both attempted to capture behavioral laughter in the lab or by using retroactive self-reports). However, neither of those studies incorporated *both* methodological innovations: use of a coding scheme and the recording of naturalistic behavior, beyond self-reports. Moreover, no prior study has examined between-group differences focusing on behavioral laughter of mothers in the natural environment.

#### Laughter as a function of cultural processes

Although laughter has not been studied before in the context of cultural differences, there is evidence showing that Latinxs value positive affectivity. For example, research has shown that Latinxs living in the U.S. report experiencing more positive emotions (such as joy, happiness, excitement) and less negative emotions (such as sadness, worry, irritation) as compared to other cultural groups (including European-Americans, Asian-Americans, Japanese and Indians) [34]. Similarly, Mexican exchange students in Canada endorse more excited states (such as elatedness, happiness) as compared to Chinese students in Canada who value more calmed states (such as serenity, restfulness) [35]. Latinx not only demonstrate positive affectivity in questionnaires, but they also conduct themselves in a manner that facilitates positive social relationships. Specifically, Latinx behave in a polite, respectful and positive way when they interact with others; they emphasize friendliness and prefer to avoid conflict by accentuating positive behaviors and deescalating negative behaviors that might lead to it [3–6]. This specific way of behaving has been defined as the cultural value of Simpatía, which has been used to describe a pattern of social interaction that characterizes people of Latin American descent [36]. In sum, Latinx value positive emotional expressivity, as measured by questionnaires, and they also behave in a manner that emphasizes positive social interactions; therefore, we can assume that in the service of fomenting positive social interactions, Latina mothers might laugh more than White-European mothers during those social interactions. This argument is further supported by research that demonstrates that laughter, in natural conversations, serves the purpose of facilitating the flow of the interaction and signals affiliation [37]. Similarly, laughter has been shown to be a stronger predictor of the quality of interactions, rather than an overall sense of positive affect [38].

In this study, we aimed to employ a novel approach and a unique focus on women, which could elucidate some of the complex relationships between psychosocial variables and overall well-being in women. Although the studies reviewed above on laughter and well-being have used different methodologies and are not void of nuances, we argue that the more opportunities mothers have to experience behavioral laughter as a function of the quality of their conversations in their everyday lives, the more likely they will experience better well-being.

## Study overview

In this study, we used as a framework the sociocultural resilience model to test if Latina cultural processes are associated with a psychological marker of well-being (i.e., behavioral laughter) and if the quality of the conversations mediate this relationship. We utilized data that was collected for a large-scale study at a research institute in the Seattle area. Latinx and White-European parents of approximately one-year-old infants were recruited, and both the infants and their mothers (as well as a few fathers) were asked to wear a digital recorder to capture their natural social behaviors and conversations. The data captured by the infants’ recorder has been transcribed, coded, and published elsewhere [39–42].

As a proxy for **cultural processes**, we considered the ethnicity of the mothers (i.e., Latina vs. White-European). We expect that, in general, Latina mothers are more likely to value collectivism and familism and that this should have a detectable effect on the way that they socialize in their everyday lives. Although including direct measures of cultural processes might be more ideal, we decided to use a strategy similar to other studies in which ethnicity was used as a proxy for cultural processes [2, 3, 11, 20–22].

To define the cohesiveness of **social networks**, we used a similar approach as Mehl and colleagues [43]. In their study, the authors asked college American students to wear a digital recorder to capture snippets of natural sounds as participants went about their lives. They then asked coders to examine snippets in which conversation occurred to classify each as either small talk (defined as uninvolved, banal conversation) or substantive (defined as involved, meaningful conversation). The authors then quantitatively assessed the participants’ well-being using a combination of self-reported questions about life satisfaction and a single item of happiness with reports from two to three other informants. The authors found that substantive conversations were positively related to well-being, whereas small talk was unrelated to well-being.

In this investigation, we examined the mothers’ quality of conversations (i.e., small talk and substantive conversations) within two social networks (i.e., conversations with the father of the infant vs. conversations with other adults). Mothers’ quality of interactions not only include conversations with relatives, friends, and acquaintances, but also the conversations with the father of their child. Since our data uses a naturalistic approach, it gives us the unique opportunity of exploring the role of different social networks as mothers go about their lives.

As a **marker of well-being**, we used the percentage of time mothers laugh during their conversations with adults. We focused on laughter that occurs during social interactions with adults because laughter has been defined as a social signal that transpires more often when people are interacting with others than in the presence of other stimulating media (such as television, radio or books) [44, 45]. Furthermore, since our goal is to define the cultural processes associated with laughter, we argue that those cultural processes (such as Simpatía and positive expressivity in Latinas) are relevant only when laughter occurs while interacting with others adults and not relevant when laughter occurs in other settings. Finally, since our goal is to observe if levels of behavioral laughter change according to the quality of the conversations (i.e., substantive conversations vs. small talk), it is important to focus on settings where these conversations are more likely to occur (i.e., while interacting with adults).

**Fig 2:**
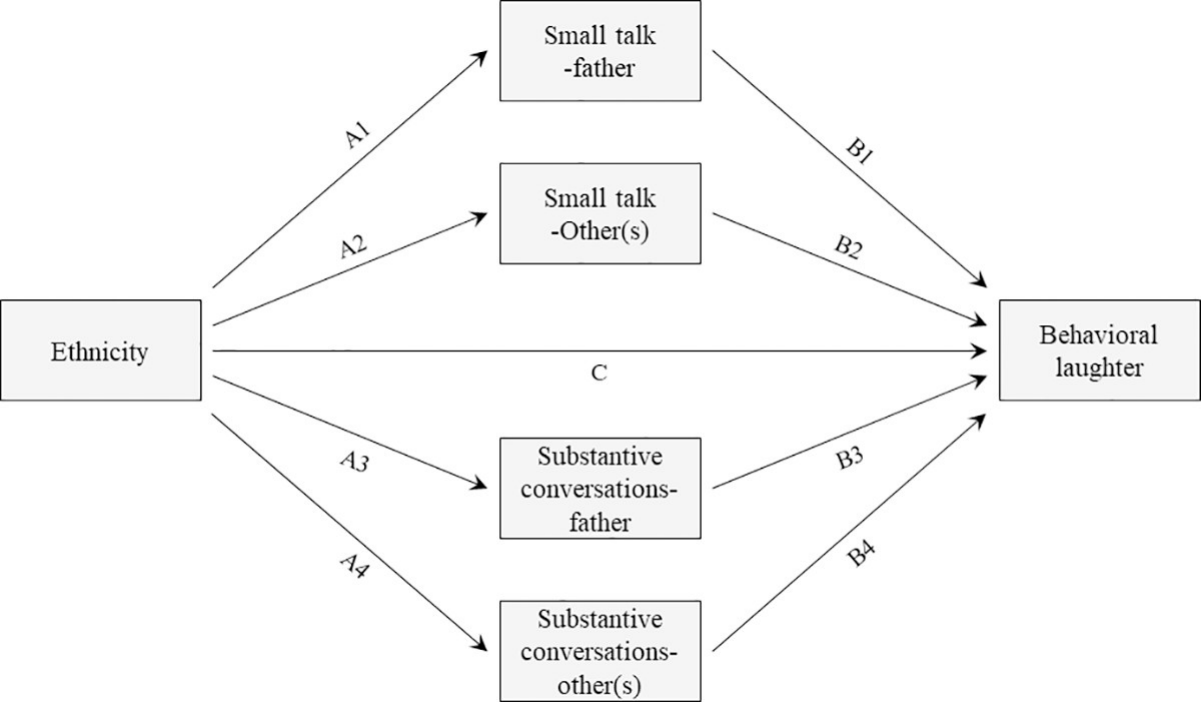
Sociocultural resilience model tested in this study. This model illustrates how four conversation style variables may mediate the association between ethnicity (i.e., being a Latina mother vs. a White-European mother) and behavioral laughter.

In sum, we used the sociocultural resilience model [1] presented in Fig 1 as a framework for the model shown in Fig 2. The model in Fig 2 includes the effect of ethnicity on behavioral laughter, and explores whether the quality of the conversations mediate the relationship between ethnicity and behavioral laughter. We test four mediators: Latina and White-European mothers’ use of small talk with the father, and with other adult(s) (i.e., small talk-father, small talk-others(s), respectively) and their use of substantive conversations with the father and with other adult(s) (i.e., substantive conversations-father, substantive conversations-other(s), respectively). Next, we make some expectations; however, given that there is no previous work that directly investigates the quality of conversations within different social networks (e.g., the father vs. other adults), we did not have specific expectations for the role this might have as a potential mediator. Therefore, the role of different social networks is tested in an exploratory manner.

### Expectations for the pathways shown on Fig 2

#### Ethnicity and quality of the conversations (Path A)

We tested the relationship between ethnicity and small talk-father (Path A1); ethnicity with small talk-other(s) (Path A2); ethnicity and substantive conversations-father (Path A3); and ethnicity with substantive conversations-other(s) (Path A4). We expected an association between ethnicity and substantive conversations, and no association between ethnicity and small talk. Specifically, we hypothesized that being a Latina mother would be associated with substantive conversations. This expectation is consistent with the cultural value of familism, which suggests that Latinx have strong identification and attachment with their nuclear family and extended families [7–9] and tend to live close to their relatives [11]. Thus, if Latina mothers have more opportunities to interact with close others, then they would likely engage in more substantive interactions than White-European Americans.

#### Quality of the conversations and behavioral laughter (Path B)

We tested the relationship between small talk-father and behavioral laughter (Path B1); small talk-other(s) and behavioral laughter (Path B2); substantive conversations-father and behavioral laughter (Path B3); and substantive conversations-other(s) and behavioral laughter (Path B4). We expected that substantive conversations would be correlated with behavioral laughter, independent of the group. In contrast, we expected that small talk would be unrelated to behavioral laughter, independent of the group. This expectation was drawn from Mehl and colleagues’ study [43] where they reported a positive association between substantive conversations and well-being, but small talk was not associated with well-being. Although behavioral laughter was not directly assessed by Mehl and colleagues, we assume that laughter may occur more frequently when mothers have substantive conversations while interacting with adults indicating affiliation and closeness [37,38], and if mothers have more opportunities to have substantive conversations and laugh during those conversations then they will experience an overall sense of well-being [30, 31].

#### Ethnicity and behavioral laughter (Path C)

We tested the relationship between ethnicity and behavioral laughter (Path C). Consistent with the literature on the Latinx health paradox [1], we expected that being a Latina mother would be positively associated with behavioral laughter. Although behavioral laughter has not yet been examined as a Latinx well-being advantage, being a Latinx has been associated with positive psychological health outcomes [19–24]; and with Latinxs’ cultural processes associated with positive expressivity [3–6, 37, 38]. Furthermore, laughter has been linked to positive physical health outcomes [28], and well-being benefits [30].

## Method

### Participants

Fifty-four families were recruited as part of a larger study at a research institute in Seattle, WA. Of those, 26 families identified themselves as Latinx and 24 families identified themselves as White-European. One family identified themselves as African-American, so they were not included in the analyses. Participants gave informed consent to participate in the study. The University of Washington Institutional Review Board approved this project.

Since the participants had the option of not responding to the demographic questions, there is missing question data for some of the participants. For each question, we indicate the number of participants that provided the information.

### Latinx families

Twenty-six Latinx families participated in this study. Of those, only 25 mothers provided their age (M = 32.14, *SD* = 4.94) and only 25 fathers provided their age (M = 34.57, *SD* = 7.82). Twenty-four families provided information about their income. Twenty-four families provided information about their income (average annual income = 50,000–75,000 dollars, minimum 20,000–25,000 and maximum = 100,000–200,000). All families had an infant of about one year of age. Twenty-four families reported that the infant lived at home with the mother and father and two reported that the infant lived only with the mother. Furthermore, seven families had one other older child living at home and five families had two older children living at home. Two families reported having grandparents living at home and one family reported having an uncle living at home (for cultural and language characteristics of the families, see information below).

#### Latinx families’ language and cultural characteristics

Twenty-three of the 26 participants responded to a language background questionnaire.

Regarding the mothers, one mother was born in Peru, one in Puerto Rico, one in El Salvador, two in Venezuela, three in Colombia, seven in Mexico, and eight in the United States. Including the mothers who indicated that they were born in the United States, the average number of years that the mothers had been living in the United States was 16.20 years (*SD* = 9.68). Ten mothers preferred to use Spanish in daily life, four preferred English, and nine preferred both languages.

Regarding the fathers, one father was born in Peru, one in El Salvador, one in Guatemala, one in Venezuela, one in Ecuador, two in Colombia, two in Puerto Rico, seven in Mexico, and seven in the United States. Including the fathers who indicated that they were born in the United States, the average number of years that the fathers had been living in the United States was 14.79 years (*SD* = 10.33). Thirteen fathers preferred to use Spanish in daily life, five preferred English, and five preferred both languages.

### White-European families

Twenty-four White-European families participated in the study. Mothers’ average age was M = 35.31, *SD* = 6.04. Only 22 fathers provided their age (M = 36.43, *SD* = 5.86). Twenty-one families provided information about their income (average annual income = 50,000–75,000 dollars, minimum 25,000–50,000 and maximum = 200,000 and above). All the families had an infant of about one year of age. Twenty-four families reported that the infant lived at home with the mother and father. Four families had one other older child living at home and one family had two other children living at home. One family reported having the grandparents living at home.

### Data collection and data preparation

#### Data collection

Families received two digital language processors (DLPs) and an armband to hold the DLP. Of the 26 Latinx families, 25 mothers wore the recorder and one father wore the recorder; and of the 24 White-European families, 21 mothers wore the recorder and three fathers wore the recorder. They were instructed to record eight continuous hours each day for four days (including two weekdays and two weekend days), yielding approximately 32 hours of recorded audio from each family. The participant who wore the recorder was also asked to complete a daily activity diary, noting the most relevant activities for each day. In the diary, the participant was asked to enter the day of the week, the date, and what they were doing in each hour of the day (e.g., at home having lunch, at a friends’ house at a playdate, having dinner, etc.) for each of the four days that they wore the recorder.

#### Data preparation

The audio data were transferred from the DLP to a computer and analyzed by LENA software. The software was used to analyze language input and to efficiently locate intervals with the language activity of interest (i.e., adult speech) in each participant’s dataset to use for further conversation-quality analysis. The LENA algorithms produced a total adult word count across all four days for each participant in the study. The accuracy of these values for the English language has been estimated in previous studies [46, 47]. For the Spanish language, Weisleder and Fernald’s [48] research team transcribed 60-minute samples from ten participants in their study. Their analysis of these transcriptions showed a strong positive correlation (i.e., *r* = .80) between automated estimates and transcribed word counts.

We then used the same approach as other studies [39–42]. Specifically, using the LENA Advanced Data Extractor Tool (ADEX) each participant’s dataset of recorded audio was segmented into 30-second intervals, to automatically estimate an adult word count for each interval. For example, an eight-hour recording yields approximately 600–960 intervals with adult word counts after the data are segmented into 30-second intervals. Intervals with no adult words were removed, and 50 intervals were selected from the remaining intervals across the entire day (i.e., 200 across the four days), chosen from those with the highest adult word counts. Although the adult word count is an estimate done by the software, intervals were selected based on adult word count to ensure that there is language activity that will allow coding of social behaviors. Using this approach, we avoided selecting intervals for coding where there was no social activity, but only silence or noise (e.g., the adult is alone at home; the adult wasn’t wearing the recorder). Furthermore, we segmented the data based on 30-second intervals, since other studies have assessed social behaviors in adults from 30-second snippets [2, 49, 50] and was employed in previous studies with infants and children [39–42]. The average word count across the selected intervals for the Latina mothers was 77.77 (*SD* = 25.48) and for the White-European mothers was 84.72 (*SD* = 37.66). No significant differences were found across the groups (t = .78).

#### Compliance

Ideally, the final data set would include for coding a total of 200 intervals for each participant; however, sometimes fewer intervals were selected if data was not available for participants (e.g., participant did not wear the recorder). On average, 190.42 (*SD* = 17.28) intervals were coded for the Latinx families and 184.25 (*SD* = 33.05) for the White-European families. No significant difference was found between the number of intervals coded across groups (t = .84, *p =* .40). Across all 26 Latinx families a total of 4,951 intervals were coded and across all 24 White-European families, a total of 4,422 intervals were coded.

Intervals were excluded from analyses if there were problems noted during coding of the recording (e.g., participant wasn’t wearing the recorder, excessive noise; M = .03, *SD* = .03 for the Latinx families; M = .04, *SD* = .05 for the White-European families). An average of 184.38 (*SD* = 17.71) intervals for the Latinx families and an average of 177.46 (*SD* = 33.17) intervals for the White-European were included in the analyses. No significant difference was found between the number of intervals included for analyses between groups (t = .93, *p =* .36).

As a behavioral marker of obtrusiveness, research assistants coded how often the participants mentioned the recorder to other people during recording. On average, White-European families mentioned the recorder 2.73% of the time (*SD* = 1.71%) and on average Latinx families mentioned the recorder 1.91% of the time (*SD* = 1.58). This replicates past studies showing that behavioral data collection using recorders operates unobtrusively [51, 52].

### Coding the quality of the conversations

We identified variables of interest using the Social Environment Coding of Sound Inventory (SECSI, e.g., [2]). We adapted the SECSI for the goals of this investigation and used the following five categories: (1) mother-father—the mother is talking to the father; (2) mother-other(s)—mother is talking to other adult(s); (3) small talk—uninvolved conversation and only trivial information is exchanged (4) substantive conversations—the conversation is involved and meaningful information is exchanged; there is dialogue, gossip, sharing of feelings, and (5) laughter-overall—the mother is laughing during the interval; there is an audible chuckle and/or loud laughter (includes laughter while talking to others or laughter while interacting with infant). See Table 1 for examples of conversations that were characterized by the coders as small talk and as substantive conversations.

**Table 1:** Examples of small talk and substantive conversations.

Example	Small talk	Substantive conversations
**1.**	*That barbeque sauce your brother used is sure good*. *Is it just the craft honey barbeque sauce*?	*In the USA fifteen children and teens are killed each day by guns*. *In some regions more then more young men are killed by guns then by car crashes*. *Almost two-thirds of all suicides involve guns*. *Suicide is the third leading cause of death for teens and young adults*. *Every three hours a teen takes their life with a gun*.
**2.**	*Deep fried pancakes*? *Oh that's so gross*! *That would be donuts*. *That's probably why you don't like them huh*?	*Well I was just reading again from a couple of doctors*, *that you really want to give your baby a good start*. *They are saying two years now*. *They are more and more recommending them*, *it is funny*.
**3.**	*Yeah*, *it is raining pretty hard*, *I was wondering how you all were staying dry*, *you must have been in the community circle for the worst of it*	*If you get put on a jury and let us say it is a murder trial it can go for months*. *You would have to go everyday and they pay you like ten dollars a day*.
**4.**	*Okay I will get there early*! *Things take fourteen times longer when kids are involved*.	*We were pulling up to the airport and she goes 'Oh*, *I am feeling sick'*. *And instead of getting off at the airport until she felt better*, *for an hour and a half we have been riding in the bus*, *she was sitting sideways with her eyes closed*.
**5.**	*Did you see my email about the xxxx gardening*? *it is kind of neat huh*?	*I might not get a grant so it may not be an option*. *Yeah*, *but I also heard that from somebody that xxxx are not giving money right now*.
**6.**	*Excuse me*, *excuse me*. *Do you work here*? *I just need a white balloon*. *The white*. *The plain one*. *The white*. *This one right here*.	*I don't know if you talked to her last night*. *About the whole xxxx thing*. *She said that she had just gotten into an argument with him in one of the level chat channels*.
**7.**	*Oh*, *I just came this way instead today*. *There is a lot of traffic*. *Um*.	*My parents have a Siamese before I was born*. *And I think they had to give it my grandparents because they thought I was allergic*. *and But I think he lived until*, *I was definitely in college when he died*.
**8.**	*I put a slice of American cheese and then I put some regular cheese*	*Well*, *I kind of have noticed that he was avoiding me last time when he was home*, *but there is this one time when he said something to me and I am controlling it and I was tired*
**9.**	*I think we are going to take off and go to the store for a little bit*	*In Chile*, *actually where my parents live the seasons are very similar to here*, *just the opposite of here*. *So they come here every August*, *or July and they are so happy to get out of the rain and the cold and we go there usually in December*, *January*
**10.**	*The dishes in the dishwasher are clean*	*I am not a student so I am in an intermediate position when you are still under somebody*. *So you are not on your own*. *So you are in their lab working on their project*, *and you are supervised by them*.

Three Spanish-English bilingual and four English monolingual coders, who were blind to the hypotheses of this study, were trained for coding and were tested independently by using a training recording sample from a White-European participant (used to evaluate inter-coder reliability). Table 2 shows the intra-class correlation for the five categories used in the analysis. Although the intra-class correlation for small talk was not optimal (i.e., .60), the average intra-class correlation across the five categories was .83—indicating effective training and reliable coding—based on a two-way random effects model (ICC [2, k]; [53]).

**Table 2:** Conversation style variables for latina and white-european mothers: Descriptive statistics and reliabilities.

		Latina mothers		White-European mothers			
		N = 26		N = 24			
	Inter-coder Reliability	Relative Time Use Percent Interval		Relative Time Use Percent Interval			
Categories		Mean	SD	Mean	SD	t-value (indep)	Effect size (d)
1. Mother-father	.92	22.61	12.47	21.76	8.45	.29	.08 (-.48 -.63)
2. Mother-other(s)	.93	29.40	12.41	28.33	14.71	.28	.08 (-.48 -.63)
3. Small talk	.60	46.83	21.80	43.43	33.26	.43	.19 (-.36 -.75)
4. Substantive conversations	.80	49.89	14.13	39.82	20.31	2.05*	.58 (.01–1.15)
5. Laughter-overall	.92	29.74	8.35	20.24	11.52	3.32**	.95 (.37–1.54)
Transformed Categories							
6. Small talk-father	__^a^	9.25	8.19	7.77	7.62	0.66	.19 (-.37 -.74)
7. Small talk-other(s)	__^a^	9.16	7.07	12.21	15.26	-0.89	-.26 (-.82 -.30)
8. Substantive conversations-father	__^a^	14.51	8.82	13.16	9.00	0.54	.15 (-.40 -.71)
9. Substantive conversations-other(s)	__^a^	22.59	9.68	15.88	11.41	2.25*	.64 (.07–1.21)
10. Laughter-father	__^a^	7.08	5.76	4.51	3.31	1.92	.54 (-.02–1.11)
11. Laughter-other(s)	__^a^	9.97	4.45	7.78	6.86	1.34	.38 (-.18 -.94)
12. Laughter	__^a^	16.21	6.08	11.74	8.23	2.19*	.62 (.05–1.19)

Note 1: Intercoder reliabilities were computed as intra-class correlations, ICC (2, k) from a training set of 100

intervals that were independently coded by 7 coders.

Note 2: The variable laughter includes laughter that occurs when talking to the father and/or other adult(s).

^a^ No reliability is reported because the variable is a transformation of two coded variables

* *p* < .05

** *p* < .01

Identified intervals were coded for each participant by a trained coder. Coders were provided with basic information about each interval (date, day of the week, time of day, and the time stamp of the audio recording) and the participants’ end-of-day diaries to supplement audio recordings. For example, if the coders listened an interval which occurred at 4:00 p.m. and the mother was speaking to a group of people, they used the end-of-day diary to learn what the participant was doing at 4:00 p.m. (e.g., she wrote in the diary “at a friends’ house having dinner.”) The coders listened to each 30-second interval and coded various behavior categories associated with the interval. In each 30-second interval the coders entered “YES” if the behavior of interest occurred.

### Further data transformation

To define the quality of the conversations across social networks, the categories “small talk” and “substantive conversations” were considered for analyses if the mother was speaking to the father and/or to other adult(s). These resulted in four subcategories: (6) *small talk-father—*mother is talking to the father and there is small talk, (7) *small talk-other(s)—*mother is talking to other adult(s) and there is small talk, (8) *substantive conversation-father*—mother is talking to the father and the conversation is substantive, and (9) *substantive conversation-other(s)—*mother is talking to other adult(s) and the communication is substantive.

To define laughter in accordance to the mothers’ social networks, laughter was included for analyses if the mother was speaking to the father or to other adult(s). These resulted in two subcategories: (10) *laughter-father—*mother is talking to the father and she is laughing, (11) *laughter-other(s)—*mother is talking to other adult(s) and she is laughing. Finally, to define laughter as it occurs across social networks, laughter was included for analyses if the mother speaks to any adult(s) and she is laughing. This resulted in one collapsed subcategory (12) *laughter—*mother is talking to the father and/or other adult(s) and she is laughing. We created this category to have an overall assessment of laughter, but only when laughter occurs in the context of a conversation with adults (i.e., either with the father or other adult(s)) and excluding laughter that may occur in other contexts (i.e., while taking care of the infant).

In this investigation, we focused on laughter that resulted from having bidirectional conversations with adults, therefore excluding laughter that occurred when the mother was interacting solely with the infant. We followed this approach because interactions with the infant are not necessarily bidirectional, since the infants’ level of language development is at the stage of using simple or complex babbling [39, 41]. Furthermore, conversations with the infant are difficult to characterize as either substantive conversations or small talk. Finally, the cultural processes in which Latinx caregivers interact with their infant are different from the cultural processes in which Latinx interact with other adults. For example, although Latinx value positive expressivity in their social interactions with adults [5, 6], Latinx caregivers in comparison with White-European caregivers, are less likely to consider their children conversational partners [54]. Thus, in order to be able to assess the role of laughter as a function conversational styles (i.e., substantive conversation vs. small talk), we focus only on laughter when interactions occur with other adults.

The data were then converted into relative time use estimates by calculating the percentage of valid intervals included in a specific subcategory across all coded intervals (e.g., percentage of intervals mother-father, small talk-father, etc.). For example, a relative time use estimate of 35% for the SECSI category “Mother speaks to father” indicated that for a participant with 200 intervals, this category was coded YES in 70 of the 200 coded intervals for that participant. See Table 2 for means and standard deviations for each of the raw categories and transformed categories for Latinx and White-European families.

## Results

### Initial exploratory analyses

The initial steps in analysis were: First, evaluate ethnic group effects (i.e., for Latinx and White-European families) for the raw categories and transformed categories. Second, assess the social networks effects (father vs. other(s)) and ethnic group effects (Latina mothers vs. White-European mothers) on small talk, substantive conversations and behavioral laughter. Third, observe the intercorrelation among the conversational style variables independently for each ethnic group. Finally, analyze the association between the conversational style variables and laughter independently for each ethnic group.

#### Mean differences by group

In order to examine mean differences as a function of ethnic group (i.e., Latina mothers vs. White-European mothers), we performed t-tests for each of the raw categories and transformed categories (see Table 2 the t-values and effects sizes). Results showed that for the *raw* categories the percent of coded intervals for substantive conversations was significantly higher (t = 2.05, p < .05) for the Latina mothers (Mean = 49.89, *SD* = 14.13) than White-European mothers (Mean = 39.82, *SD* = 20.31). Likewise, the percent of coded intervals for laughter overall was significantly higher (t = 3.32, p < .01) for the Latina mothers (Mean = 29.74, *SD* = 8.35) than White-European mothers (Mean = 20.24, *SD* = 11.52). For the *transformed* categories the percent of substantive conversations-other(s) was significantly higher (t = 2.25, p < .05) for the Latina mothers (Mean = 22.59, *SD* = 9.68) than the White-European mothers (Mean = 15.88, *SD* = 11.41). Likewise, the percent of laughter that occurs when talking to the father and/or other adult(s) was significantly higher (t = 2.19, p < .05) for the Latina mothers (Mean = 16.21, *SD* = 6.08) than White-European mothers (Mean = 11.74, *SD* = 8.23). None of the other categories were significantly different as a function of the ethnic group.

#### Mean differences by social networks and ethnicity

In order to learn if the mean levels for each conversational style variables and behavioral laughter change as a function of social networks and as a function of the ethnic group, a repeated measures ANOVA was completed independently for the percentage of small talk, substantive conversations and behavioral laughter. Social networks were examined as within-participants (i.e., father vs. other(s)) and ethnicity (Latina vs. White-European mothers) was examined as between-participants. Since we do not assume sphericity, we report F-values after using the Greenhouse-Geiser correction.

**Fig 3:**
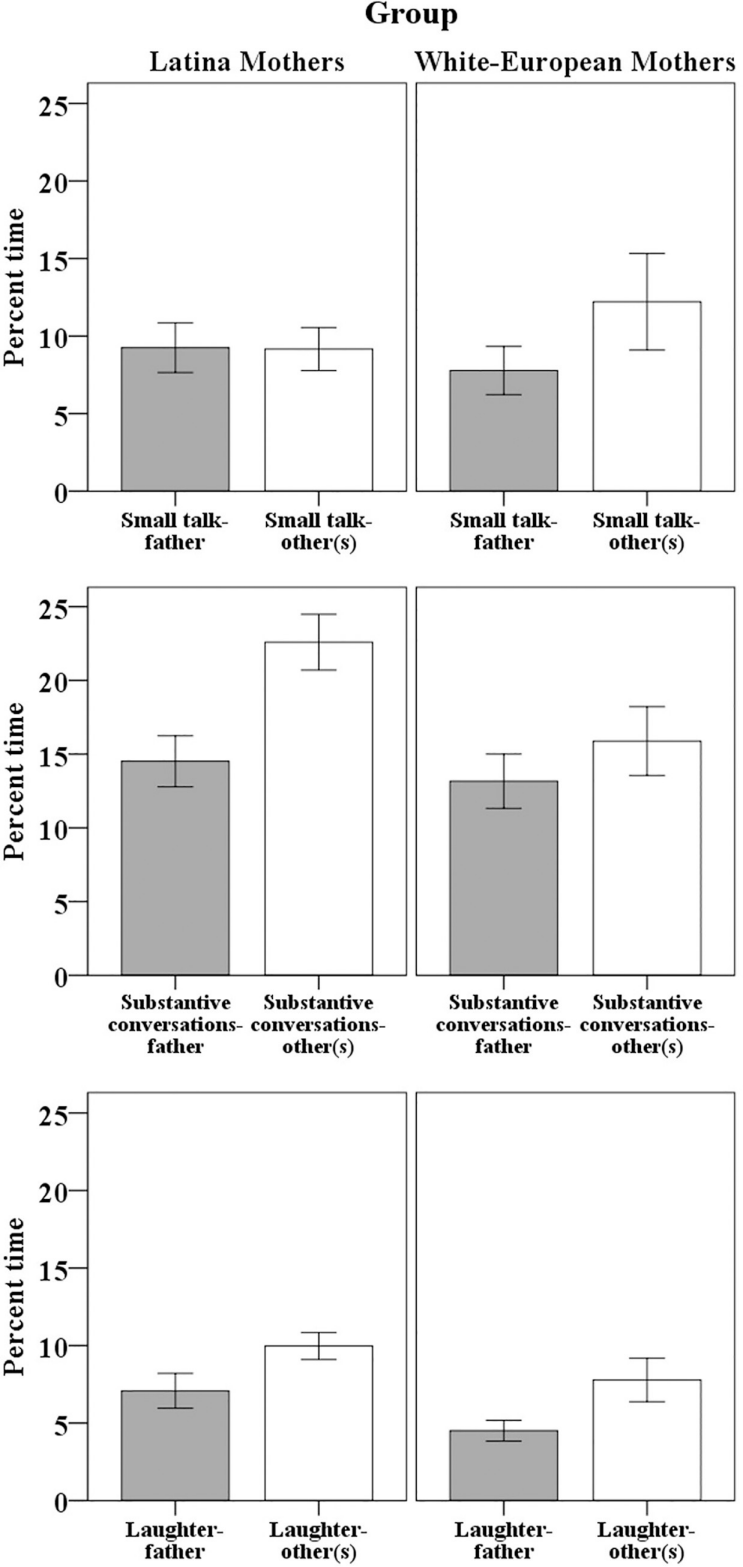
Differences for social networks and ethnicity. Main effects and interactions for social networks (i.e., father vs. other(s) and ethnicity (i.e., Latina mothers vs. White-European mothers). Error bars represent standard errors from the mean.

Concerning small talk, the social network by ethnicity interaction was not significant *F*(1, 48) = 2.80, *p* = .10, η_p_^2^ = 0.06. Furthermore, the main effects of social networks and ethnicity were not significant, *F*(1, 48) = 2.57, *p* = .12, η_p_^2^ = 0.05 and *F*(1, 48) = .10, *p* = .75, η_p_^2^ < .001, respectively (also see Fig 3).

Concerning substantive conversations, the social network by ethnicity interaction was not significant *F*(1, 48) = 1.59, *p* = .21, η_p_^2^ = 0.03. However, the main effects for social networks and ethnicity were significant, *F*(1, 48) = 6.45, *p* = .01, η_p_^2^ = 0.12, and *F*(1, 48) = 5.22, *p* = .03, η_p_^2^ = 0.10, respectively. Fig 3 shows that both Latina and White-European mothers have more substantive conversations with other adult(s) than with the father. Furthermore, Latina mothers have more substantive conversations than do White-European mothers.

Concerning behavioral laughter, the social network by ethnicity interaction was not significant *F*(1, 48) = 0.04, *p* = .84, η_p_^2^ < .001. However, main effects for social networks and ethnicity were both significant, *F*(1, 48) = 9.67, *p* = .003, η_p_^2^ = 0.17; and *F*(1, 48) = 4.56, *p* = .04, η_p_^2^ = 0.09, respectively. Fig 3 shows that both Latina and White-European mothers laugh more with other adult(s) than with the father. Furthermore, Latina mothers laugh more than White-European mothers do.

#### Intercorrelations among conversation style variables

In order to learn about the intercorrelations among the conversational style variables, we conducted correlation analyses between the four conversation style variables independently for Latina and White-European mothers. Table 3 (columns 2–4) shows that for the *Latina mothers* there was a significant correlation between small talk-others and substantive conversations-other(s). Latina mothers who have more small talk with others also have more substantive conversations with others (*r* = 0.57, *p* < .01). For *White-European mothers*, small talk-father correlated significantly with small talk-other(s), substantive conversations-father and small talk-other(s). Mothers who have more small talk with the father also have more small talk with other(s) (*r* = 0.80, *p* < .001); less substantive conversations with the father (*r* = -0.48, *p* < .05), and with others (*r* = -0.43, *p* < .05). Finally, small talk-others correlated significantly with substantive conversations-others. Mothers who have more small talk with others use less substantive conversations with the father (*r* = -0.54, *p* < .01).

**Table 3:** Correlations between conversation style variables and laughter for both Latina and White-European mothers.

	Conversation style variables				Laughter		
Latina mothers (N = 26)	Small talk-father	Small talk-other(s)	Substantive conversations-father	Substantive conversations-other(s)	Laughter-father	Laughter-other(s)	Laughter
Small talk-father	1				0.63***	-0.07	0.45*
Small talk-other(s)	0.33	1			-0.03	0.62***	0.36
Substantive conversations-father	0.33	-0.33	1		0.78***	-0.35	0.45*
Substantive conversations-other(s)	-0.16	0.57**	-0.36	1	-0.23	0.79***	0.31
White-European mothers (N = 24)							
Small talk-father	1				0.24	0.01	0.11
Small talk-other(s)	0.80***	1			-0.05	0.18	0.15
Substantive conversations-father	-0.48*	-0.54**	1		0.05	-0.18	-0.14
Substantive conversations-other(s)	-0.43*	-0.29	-0.04	1	0.06	0.65***	0.51**

Note 1: The variable laughter includes laughter that occurs when talking to the father and/or other adult(s).

* *p* < .05

** *p* < .01

*** *p* < .001

In order to learn about the association between conversational style variables and behavioral laughter, we conducted correlation analyses between the four conversation style variables and the three laughter outcomes independently for both Latina and White-European mothers. Table 3 shows that when considering laughter across different social networks (columns 6 and 7), significant correlations were found for Latina mothers between small talk-father and laughter-father (*r* = 0.63, *p* < .001); small talk-other(s) and laughter-other(s) (*r* = 0.62, *p* < .001); substantive conversations-father and laughter-father (*r* = 0.78, *p* < .001); substantive conversation-other(s) and laughter-other(s) (*r* = 0.79, *p* < .001). For White-European mothers, a significant correlation was only found between substantive conversations-other(s) and laughter-other(s) (*r* = 0.65, *p* < .001).

Furthermore, Table 3 shows that when considering laughter collapsed across social networks (column 8), for Latina mothers, significant correlations were found between small talk-father and laughter (*r* = 0.45, *p* < .05), as well as between substantive conversations-father and laughter (*r* = 0.45, *p* < .05). For White-European mothers, only the correlation between substantive conversations-other(s) and laughter was significant (*r* = 0.51, *p* < .01).

### Testing the sociocultural resilience model

**Fig 4:**
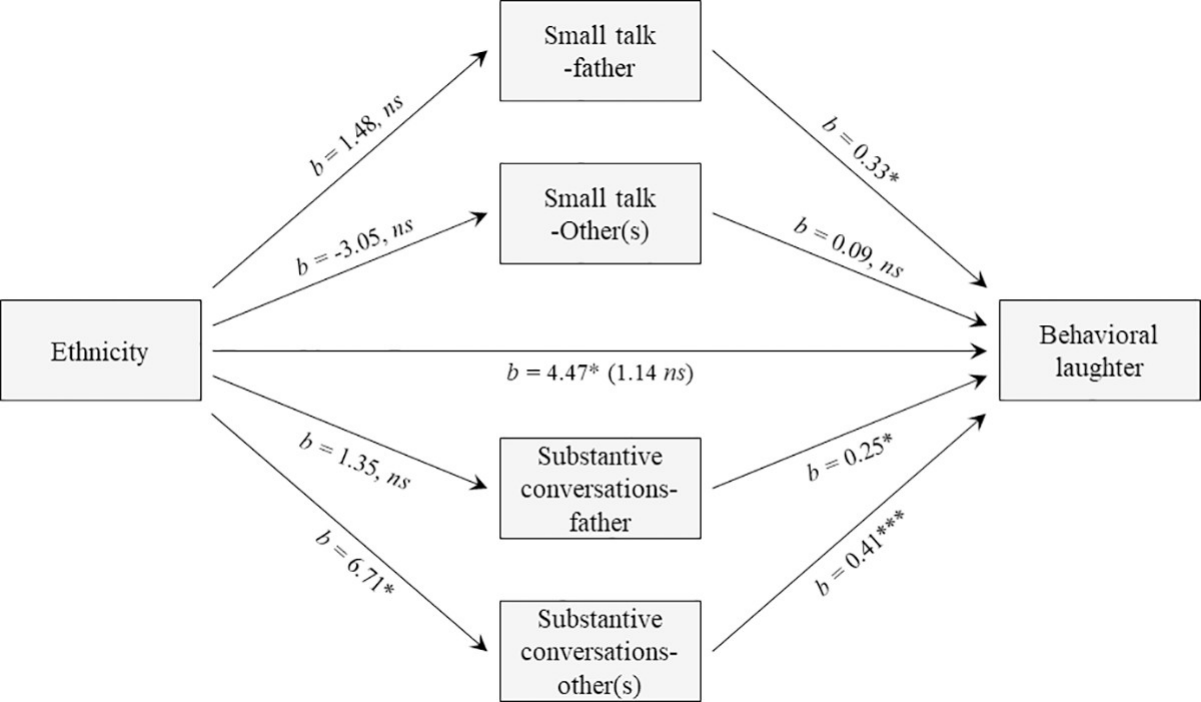
Mediation analyses. This analyses shows that substantive conversations-other(s) mediates the relationship between ethnicity and laughter. *b* = indicates the regression coefficient. * *p* < .05, ** *p* < .01, *** *p* < .001.

To apply the sociocultural resilience model to this study, we performed mediation analyses to follow the same approach that we have used in other relevant studies [39, 41]. Specifically, to investigate if the four conversation style variables mediate the association between ethnicity and laugher, we considered the variable laughter across social networks (i.e., laughter that occurs with the father and/or other adults). Ethnicity was converted to a dummy code variable and Latina mothers were given a score of “1” and White-European mothers a score of “0.” Finally, we used a multiple mediation macro by Hayes’ Process [55], with 1,000 bootstrapping re-samples. Fig 4 shows that there was a positive and significant relationship between ethnicity and laughter (Path C; *b* = 4.47, *p* = .03). Furthermore, only substantive conversations-other(s) mediated this association. Specifically, results show that there was a positive and significant relation between ethnicity and substantive conversations-other(s) (Path A4; *b* = 6.71, *p* = .03, 95% *CI* [1.70, 11.72]), and a positive and significant relation between substantive conversations-other(s) and laughter (Path B4; *b* = .41, *p* < .001, 95% *CI* [.27, .55]). In addition, the relation between ethnicity and laughter was reduced in magnitude when substantive conversation-other(s) was included in the model (Path C’; i.e., from 4.47, *p* = .03 to 1.14, *p* = .53). Substantive conversations-other(s) was deemed a significant mediator because the 95% bias-corrected confidence interval did not include zero, 95% CI [.1.01, 5.30]. The final model explained 31% of the variance of laughter (Fig 4).

## Discussion

In this investigation, we relied on the sociocultural resilience model described by Ruiz and colleagues [1] and we tested the relationship of ethnicity on behavioral laughter, and we analyzed if this association was mediated by the quality of the conversations. The quality of the conversations was assessed using a similar approach to Mehl and colleagues [43]; however, in this investigation we included different social networks where different conversations occur (i.e., with the father or with other adults). This resulted in four conversation style variables (i.e., small talk-father; small talk-other(s); substantive conversations-father; substantive conversations-other(s)). Overall, the results demonstrated that being a Latina mother relates to greater percentage of behavioral laughter and that the only conversation style variable that mediates this relationship is substantive conversations with others.

### The quality of the conversations with different social networks

In this study, we explored for the first time the role of different social networks on the quality of the conversations and on behavioral laughter. Interestingly, the results showed that substantive conversations occur more often when both Latina and White-European mothers converse with others than with the father of their child. Furthermore, both cultural groups laugh more when they converse with others than with the father. This finding could be the result of the particular characteristics of the families. All families recruited had an infant of about one year of age living at home and most of them were first-time parents. Consequently, we can imagine that mothers’ conversations with the father of their infant might involve themes regarding everyday practicalities of taking care of the child and household responsibilities (i.e., more uninvolved, banal conversation). However, when the mothers can interact with others, they will disclose more and talk about their deep thoughts and feelings. For future investigations, it would be important to observe whether deemphasizing conversations of quality and laughing less with the father is associated with the everyday struggles of taking care of an infant, and how this is related to marital satisfaction [56, 57].

### Differences on ethnicity for social networks and behavioral laughter

The relation between the quality of the conversation, social networks, and behavioral laughter was different for each ethnic group. For White-European mothers, findings were comparable to the findings from Mehl and colleagues study [43]. Small talk with the father and other adult(s) was unrelated to behavioral laughter. However, substantive conversations with other(s) was related to behavioral laughter. Interestingly, substantive conversations with the father was unrelated to behavioral laughter. This is probably because, as was mentioned before, the families were first time parents and were overwhelmed with day-to-day responsibilities of taking care of an infant. Of interest for future investigations, however, would be to observe the degree that having substantive conversations with others, and laughing during those conversations, serves as a buffer for marital satisfaction [32, 58, 59].

In contrast, for Latina mothers, behavioral laughter occurs within each of their conversations with each of their social networks. If they talked to the father and they had small talk, they laughed; if they talked to others and they had substantive conversations, they laughed. This finding could be explained by the sociocultural processes that characterize Latinxs such as valuing positive emotions [34, 35] and behaving in ways that emphasize positive relationships by being friendly, polite, and respectful [4–6]. This finding also lends support for the Latinx health paradox [1]. Since the Latinx paradox explains that although the Latinxs in the United States experience physical health challenges (e.g., high rates of child and adult obesity [60]), and other psychosocial disadvantages (e.g., discrimination, [61]), Latinxs have greater life expectancy that other Non-Latinx groups [19]. Behavioral laughter during social interaction may bring positive health outcomes [29, 30, 32, 33] and protect against the negative effects of the physical and psychological challenges that Latinx in the United States face in their everyday lives. Future investigations must include measures of physical health to further understand the role of laughter during social interactions as a possible explanation for the Latinx health paradox.

### Applying the sociocultural resilience model

Ruiz et al. [1] proposed in their sociocultural resilience model that Latinxs’ cultural processes (e.g., familism) influence health advantages and other positive indicators of health through the activation of social networks (see Fig 1). In this study, we used ethnicity as a proxy for cultural processes and behavioral laughter as a positive indicator of well-being, and we defined social networks as the conversations of quality that mothers have with the father and with other adult(s). This led us to test the activation of four conversation style variables. By using this approach, we were able to observe which conversation styles matter for behavioral laughter. We found substantive conversations with others was the only conversation style that mediated the relation between being a Latina mother and behavioral laughter.

These findings go in agreement with Mehl and colleagues study [43] in which substantive conversations, but not small-talk, was related to self-reported well-being and happiness. This finding provides some support for the sociocultural resilience model. For example, the finding suggests that Latina mothers’ cultural processes, such as familism and collectivism, afford more substantive conversations with other(s) and results in more behavioral laughter, a process that can lead to positive well-being outcomes. Although in this study we did not assess actual cultural processes, the findings correspond with other studies that have observed the interplay of cultural processes (e.g., familism), social support and other health advantages (e.g., cortisol levels, [25]) and psychological well-being (e.g., stress, general mental health, and depressive symptoms [24]). Future studies, however, should address the following shortcomings from this study.

First, it would be informative to complement behaviors with self- reports and other-reports of well-being and happiness (as in Mehl et al. [43]); as well as more explicit assessments of acculturation [e.g., 9, 12], familism [e.g., 7, 8], and collectivism [2, 3] to confirm that these values are stronger in the Latinx.

Second, it is important to include questions of self-reported social interactions [62] to observe if Latinas’ substantive conversations are affected by the fact that they spend more time talking to close friends and relatives as compared to White-American mothers. If Latina mothers indeed spend more time talking to relatives and close friends, then that would not undermine the findings from this investigation. On the contrary, it would show that Latinx cultural processes influence the types of interactions that they choose to have, which in turn leads them to engage in more substantive conversations and therefore have more opportunities to laugh.

Finally, in this study, we used cross-sectional data to test for potential mediation. Whether cross-sectional data is suitable for analysis using Hayes’ Process [55] is a statistical debate (e.g. see [63], for more information), yet we believe it is suitable to conduct mediation analysis due to the theoretical model we used that is based on previous findings established in the literature (i.e., socio-cultural resilience model [1]). However, to further test this model, future research should employ longitudinal and experimental study designs.

### Other future directions, strengths, and limitations

Although women’s physical and psychological health has been extensively reported in the literature, there are no studies that have focused on the interplay of being a woman and how that relates to everyday conversations with different partners and how these interactions positively moderate or mediate well-being outcomes. Future studies are necessary, however, to understand how women benefit from those social interactions in ways that help them overcome different obstacles regardless of their cultural background. For example, the studies by Campos and colleagues [24] demonstrate that women from different ethnic backgrounds (i.e., Latinas, Asian and European-American) report higher familism and social support, but also poorer mental health than men. Familism, however, was indirectly positively associated with better psychological health via social support; suggesting that for women the quality of their relationships can protect them from experiencing poor physical and mental health.

Another future direction would to assess how conversations of quality help women to manage the challenges of work and family roles [64], or first-time motherhood and marital satisfaction [56, 57]. Unfortunately, our study cannot respond to any of these questions, but the findings from this investigation highlight how the way women interact in their everyday lives can have a social benefit that may buffer everyday problems of being a women. In this regard, it is also important to include fathers’ everyday interactions to observe how these types of interactions benefit equally or differently women and men.

Future studies would benefit from including a sample of mothers with older children or a sample of women in a committed relationship with no children. This would shed light on whether, and how, substantive conversations with the father were affected by the characteristics of the families in this particular study. That is, it would help illuminate the distinction between the importance of having substantive conversations within *any* social network versus having meaningful conversations with a romantic partner or other(s). It is possible, however, that conversations of quality with others affords more positive affect than conversations with romantic partners. Examining this stimulating question in the future would help disentangle the interplay of the quality of the conversations with different social networks and its effects on behavioral laughter.

One strength of this study is that we assessed a behavioral outcome using a naturalistic, observational design. This approach allowed us to bypass the use of self-reports, which have limitations in cross-cultural designs. For example, participants can overestimate or underestimate their standing on socially desirable phenomena due to cultural biases [4, 65, 66]. Despite the strengths of using observational data, our design has some important limitations. First, our variables are non-exhaustive and non-mutually exclusive; thus the conversational styles were not fully independent (see Table 3). Thus, our variables have the same shortcomings as self-reports that include different dimensions (e.g., the Big Five dimensions of personality are not fully orthogonal, for a discussion see [67]).

Second, although our goal was to focus on laughter while the mother was talking to adults, we cannot fully be certain that the selected intervals did not include laughter that occurred within the context of other stimulating situations. For example, the mother could have been laughing while watching TV, but also be talking to an adult within a 30-second interval. In addition, we did not code for shared laughter (i.e., laughter from the mother and other conversational partners). It is possible that the findings from this investigation is reflecting that substantive conversations is associated with shared laughter more so than unshared laughter [32]. Future studies are necessary to observe if mothers’ well-being is associated to shared laughter and to the likelihood of laughing across situations (e.g., laughter while interacting with others, while interacting with their children, while reading or while watching TV), independently of the types of conversations they have with other adults. Unfortunately, our data cannot assess these novel research questions. Qualitative analyses, however, taking into consideration more time would allow one to capture more emotional and descriptive nuances within a conversation [68].

Finally, due to the constraints of recruiting willing families with specific characteristics (e.g., Latinx families and White-European families) for an observational study, this investigation is not immune to the limitations of carrying out large-scale observational studies. For example, the study sample size is small, few fathers used the recorders, and some participants chose not to respond some of the sociodemographic questions (e.g., annual income, age, etc.). Furthermore, the samples are not equivalent in all sociodemographic variables such as family makeup and socioeconomic status. Additionally, the sample did not allow us to observe if effects change across Latinx groups. For example, previous studies questioned the applicability of the Latinx Health Paradox to Latinx from Puerto Rico [69]. Due to these limitations, the findings from this investigation need to be considered with caution, and it is important to replicate the basic findings with other similar samples [70].

## Supporting information

S1 Data Demographics and tested variables. (SAV)
